# Triplication of the *PCDH19* Gene as a Novel Disease Mechanism Leading to Epileptic Encephalopathy Resembling Loss-of-Function Pathogenic Variants

**DOI:** 10.3390/genes15101312

**Published:** 2024-10-12

**Authors:** Alba Gabaldón-Albero, Patricia Smeyers, Sara Hernández-Muela, Mónica Roselló, Carmen Orellana, Sandra Monfort, Silvestre Oltra, Francisco Martínez

**Affiliations:** 1 Translational Research Group in Genetics, La Fe Health Research Institute, 46026 Valencia, Spain; alba_gabaldon@iislafe.es (A.G.-A.); rosello_mpi@gva.es (M.R.); orellana_car@gva.es (C.O.); monfort_san@gva.es (S.M.); oltra_jua@gva.es (S.O.); 2 Pediatric Neurology Section, Hospital Universitario y Politecnico La Fe, 46026 Valencia, Spain; smeyers_pat@gva.es (P.S.); hernandez_sarmue@gva.es (S.H.-M.); 3 Genetics Unit, Hospital Universitario y Politecnico La Fe, 46026 Valencia, Spain

**Keywords:** PCDH19 clustering epilepsy, early-onset epilepsy, gain-of-dosage genetic variant, uniparental isodisomy

## Abstract

Background/Objectives: Developmental and epileptic encephalopathy 9 (DEE9) (MIM #300088) affects heterozygous females and males with somatic pathogenic variants, while male carriers with hemizygous *PCDH19* pathogenic variants are clinically unaffected. There are hundreds of pathogenic single nucleotide variants in the *PCDH19* gene reported in the literature, which lead to the loss of function of the PCDH19 protein. To date, no phenotypes associated with overexpression or copy number gains have been described in this gene. Methods and results: We present a female patient with a de novo triplication in the Xq21.3–q22.1 chromosomal region, which includes the *PCDH19* gene, which implies an unbalanced dose gain. This patient displayed a phenotype of epileptic encephalopathy compatible with DEE9. By comparison, another male patient with a similar duplication showed mild developmental delay and autism but never developed epilepsy. Conclusions: Here, we propose the dose gain of *PCDH19* as a new pathogenic mechanism that results in a phenotype similar to that found in patients with loss-of-function variants in *PCDH19*, when present in a heterozygous state.

## 1. Introduction

Developmental and epileptic encephalopathy 9 (DEE9) (MIM #300088), also known as Protocadherin 19 (PCDH19) clustering epilepsy, is an X-linked disorder caused by heterozygous pathogenic variants in the *PCDH19* gene. DEE9 is a distinct childhood-onset epilepsy syndrome. It is clinically characterized by early onset seizures, with an average age at onset of 10 months, ranging between 2 months and 3 years. The seizures usually appear as febrile and afebrile clusters. Mild-to-severe intellectual disability is common, together with psychiatric features such as autism spectrum disorder (ASD), hyperactive and/or attention-deficit disorder (ADHD), and behavioral disturbances [1,2,3].

The *PCDH19* gene locates in Xq22 and codifies an isolated (non-clustered) delta protocadherin. Protocadherins are cell adhesion molecules involved in cell-to-cell interactions such as adhesion, cytoskeleton dynamics, neural maturation, and migration. PCDH19 is expressed in neurons and glial cells during brain development and adulthood and is essential for calcium-dependent cell-to-cell interaction and adhesion [1,2,3,4]. Over 175 distinct pathogenic variants have been reported so far, showing a wide diversity from missense substitutions to complete gene deletions. All *PCDH19* pathogenic variants are believed to cause partial or complete loss of protein function. Alteration in the protein function can be expressed in several ways, among which is the alteration in the concentration at the cell surface [5]. Regarding the inheritance pattern, the DEE9 affects heterozygous women and post-zygous mosaic men, while hemizygous men are asymptomatic carriers. The random X-chromosome inactivation in women or the presence of post-zygotic pathogenic variants of the X chromosome in males both result in functional mosaicism, which is thought to be responsible for the disease. The cellular interference model aims to explain this inheritance pattern. This theory assumes that the presence of mosaicism, that is, the mixture of PCDH19-positive and PCDH19-variant cells in the developing brain of the same individual, results in a presumed perturbation of normal cell–cell interactions, ultimately resulting in abnormal circuitry that predisposes one to epilepsy [6]. This model is supported by functional studies and animal models [4,5,7]. In addition, this theory also explains the same pattern of inheritance of another X-linked genetic disease, the craniofrontonasal syndrome (CFNS) caused by loss-of-function variants in the *EFNB1* gene [8].

To date, no phenotypes associated with overexpression or increased copy number gains have been reported in the *PCDH19* gene. However, recently, some missense variants have been reported to behave as gain of function, showing a greater impairment of neuronal differentiation in heterotypic conditions [9]. Accordingly, we hypothesize that the unbalanced dosage gain in *PCDH19* results in a phenotype similar to that found in patients with loss-of-function variants.

## 2. Materials and Methods

DNA was obtained from peripheral blood leukocytes using standard methods. The karyotype was performed following the standard procedure. The array studies were performed with the Affymetrix CytoScan 750 K SNP array (ThermoFisher Scientific, Waltham, MA, USA), following the manufacturer’s recommendations for performance and quality tests. The results were analyzed with the Affymetrix Chromosome Analysis Suite v.3.1 software as recommended by the manufacturer. Clinical interpretation was carried out as previously reported [10].

Optical genome mapping: Ultra-high molecular weight (UHMW) DNA was extracted from peripheral blood samples following the manufacturer’s protocols (Bionano Genomics, San Diego, CA, USA). Briefly, mononuclear cells were digested with Proteinase K and RNAse A. Then, DNA was precipitated with isopropanol and bounded with a nanobind magnetic disk. Bounded UHMW DNA was resuspended in the elution buffer and quantified with Qubit dsDNA assay kits (Thermo Fisher Scientific). DNA labeling was obtained according to the kit protocol (Bionano Genomics). Then, 750 ng of purified UHMW DNA was labeled with DLGreen fluorophores using Enzyme 1 (DLE-1) reactions. The labeled DNA was loaded on a Saphyr chip (Bionano Genomics) and run on a Saphyr instrument (Bionano Genomics). The de novo genome map assembly was performed using BionanoSolve™ (Bionano Genomics), and structural variants were called against the human reference GRCh37/hg19 assembly. The data were analyzed with Bionano Access™ and Bionano Tools™ on Saphyr Compute Servers (Bionano Genomics).

The inactivation pattern of the X chromosome was studied, as reported elsewhere [11], with minor modifications. Briefly, two genomic DNA samples were digested with the methylation-sensitive enzyme Hpa II or with Rsa I (as control) (New England Biolabs, Ipswich, MA, USA). Once digested, the microsatellite genetic marker located in the promoter region of the AR gene, hypermethylated on the inactivated X chromosome in each cell, was characterized by PCR amplification and fragment analysis in the ABI-PRISM3130 analyzer (ThermoFisher Scientific).

## 3. Results

### 3.1. Case Reports

#### 3.1.1. Patient 1

**Figure 1:**
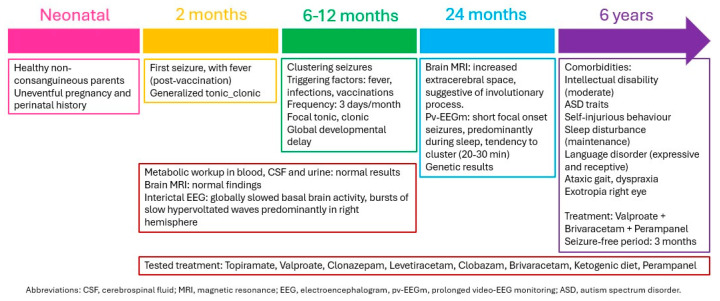
Schematic evolution of clinical findings of patient 1.

A female patient was born from healthy non-consanguineous parents with no relevant perinatal history. She presented her first seizure at two months of age, after receiving a vaccination (Figure 1). The seizures during infancy were focal with secondary generalization and appeared in clusters of less than 30 min with a frequency of 3 days per month. Although some were induced by vaccination or fever, she also displayed afebrile seizures, which were predominant during late infancy and early childhood. Focal seizures remained the main type of seizure with clustered focal tonic and hypermotor seizures with staring, behavioral arrest and moaning. No status epilepticus developed. The evolution was refractory despite several antiseizure medications including topiramate, clonazepam, levetiracetam, clobazam, and a ketogenic diet. The current therapeutic scheme at 6 years old includes valproate, brivaracetam, and perampanel, achieving a 3-month seizure free period. As comorbidities, she presents a moderate intellectual disability, autistic traits, self-injurious behavior, sleep disturbance, expressive and receptive language disorder, ataxic gait, dyspraxia, and exotropia of the right eye. 

An interictal EEG showed globally slowed basal brain activity, bursts of slow hypervolted waves predominantly in the right hemisphere, and no photosensitivity was detected. An ictal EEG reported brief clustered seizures more frequent during sleep, of focal onset, with some starting in the right posterior temporal region and propagation to the left hemisphere. The metabolic workup from blood, cerebrospinal fluid, and urine was normal. A brain MRI found no structural anomalies.

#### 3.1.2. Patient 2

The proband is a 5-year-old boy, the second child of a couple of healthy unrelated parents; his 9-year-old sister is healthy. He had no relevant perinatal history. At 9 months of life, harmonic hypogrowth together with delayed psychomotor development were noticed. The birth weight and length were in the fourth percentile, and the head circumference was in the 30th percentile, with subsequent progressive flattening of the growth curves, although not stagnation. Malabsorptive causes were ruled out, and endocrinological studies did not suggest a growth hormone deficiency. Currently, all the somatometric measurements are 3 standard deviations below the mean. Regarding psychomotor development, he reached sitting at 9 months, free walking at 20 months, with no significant motor difficulties thereafter, and delayed language development; from the first two years of life, he showed autistic traits. At present, the findings are suggestive of Autistic Spectrum Disorder grade 1. He presented a generalized epileptic seizure in the context of symptomatic hypoglycemia. No electroencephalographic alterations were detected. A brain MRI found a 3 × 1 mm cyst in the Rathke’s pouch pars intermedia.

### 3.2. Genetic Studies

#### 3.2.1. Patient 1

**Figure 2:**
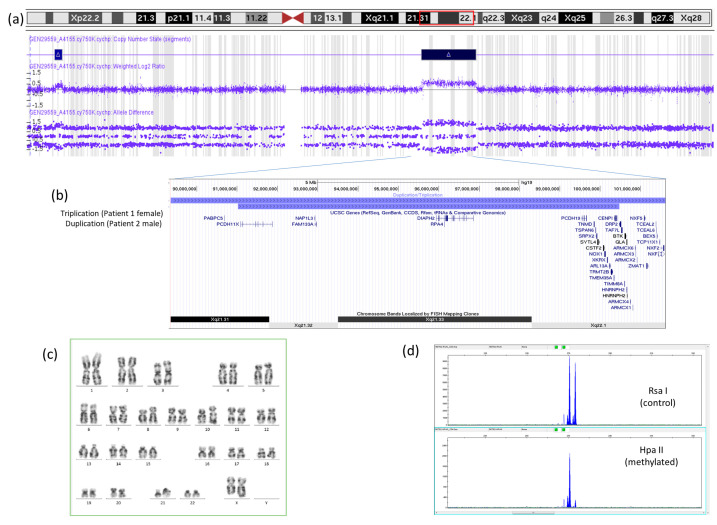
(**a**) The triplication of 12.2 Mb of the chromosomal region Xq21.3–q22.1 (chrX:89,355,579–101,615,553; Hg19) found in patient 1. Note the long run of homozygosity distal to the triplication (two SNP tracks instead of three), which results in a rare post-zygotic complex rearrangement that led to uniparental isodisomy. In addition, the three SNP tracks in the lower panel indicate that the triplication shows a 2 + 2 configuration, with two maternal and two paternal copies. Both the red box and the blue bar with a triangle inside denote the triplicated region. (**b**) The triplicated segment included 37 coding genes, of which only *PCDH19* has been associated with a compatible clinical phenotype. The duplication of patient 2, with short stature and mild intellectual disability, is also shown. (**c**) The karyotype of patient 1, confirming the in situ location of the triplication. (**d**) The pattern of inactivation of the X chromosome in blood cells showed a preferential but not complete inactivation of the maternal X chromosome.

The clinical exome sequencing failed to show variants of clinical relevance. However, in the oligo-SNP genomic array, a de novo triplication of 12.2 megabases (Mb) of the chromosomal region Xq21.3–q22.1 was found (chrX:89,355,579–101,615,553; Hg19). The triplicated segment included 31 coding genes, of which only *PCDH19* has been associated with a compatible clinical phenotype. This implied an unbalanced dose gain in the *PCDH19* gene (Figure 2).

In addition, a loss of heterozygosity was also observed from the triplicated region to the end of the long arm of the X chromosome (Xq22.1–qter). This finding highly suggested that the triplication arose as a result of a complex rarely reported rearrangement driven by break-induced replication that led to partial uniparental isodisomy [12,13]. It is worth noting that the loss of heterozygosity indicates that the chromosomal rearrangement necessarily occurred post-zygotically, during the early stages of development following fertilization.

Additionally, a duplication of 1.7 Mb was detected in Xp22.31, which contains the *STS* gene, responsible for X-linked recessive ichthyosis. This duplication was considered as a probably benign variant.

The karyotype showed an X chromosome with additional material on the long arm and an abnormal banding pattern. Optical genome mapping showed a complex rearrangement on one X chromosome with a tandem duplication of the Xp22.31 region, a triplication of the Xq21.3–q22.1 region that is in an inverted orientation, and a broad region down to the end of the long arm that exhibits loss of heterozygosity. 

The pattern of inactivation of the X chromosome in the blood cells showed a preferential but not complete inactivation of one of the X chromosomes in a proportion of 98:2. The X-inactivation pattern in neuronal cells, reflecting a functional mosaicism, is however unknown.

#### 3.2.2. Patient 2

Clinical suspicion of Silver–Russell was not confirmed by methylation studies, which also ruled out other imprinting disorders such as Temple or Kagami–Ogata syndromes. On the genomic array, a 9.45 Mb duplication was detected in the chromosomal region Xq21.31–q22.1 (chrX:91,023,673–100,477,772; hg19). This duplication contains 17 genes, of which only the *PCDH19* and *SRPX2* genes have been related to neurodevelopmental disorders, while for the *CSTF2* gene, this relationship is provisional. The duplication fully overlaps with the triplication of Patient 1, as well as partially with two presumed pathogenic duplications associated with developmental delay/intellectual disability affecting male patients in two unrelated families, as previously reported [14,15]. On the other hand, no benign duplication of the interval has been reported in healthy controls (Database of Genomic Variants). The study of the mother allowed us to determine that the mother is a healthy carrier of this duplication. X-inactivation studies in this carrier mother showed nonrandom X chromosome inactivation.

## 4. Discussion

The DEE9 or Protocadherin 19 (PCDH19) clustering epilepsy, is an X-linked disorder that displays specific clinical characteristics, as reviewed above. Patient 1 presents a phenotype highly compatible with DEE9 given the characteristics (see Figure 1): female patient, onset in infancy, clustered febrile and afebrile seizures, mainly focal and hypermotor focal seizures with affective symptoms, refractory to antiseizure treatment, with intellectual disability, behavioral problems, and ASD traits as comorbidities. DEE9 is known to be caused by loss-of-function variants in *PCDH19* in heterozygous women and post-zygous mosaic men, conditions that reflect a functional mosaicism. This implies the existence of two cell types with different PCDH19 expression, which would result in abnormal cell-to-cell interactions in the developing brain following the cellular interference model. 

In our patient, a de novo triplication of the chromosomal region Xq21.3–q22.1 was found, which implies the presence of three copies of the *PCDH19* gene in one chromosome versus one copy in the other. This unbalanced gain probably causes a neuronal mosaicism, presenting two neuronal populations with different expressions of PCDH19. Triplication may relate to an increased genic expression and thus to an increased cellular surface expression of the protocadherin. Following the theory of cellular interference, this could lead to an abnormal interaction between cells. We do not know the relevance of being a triplication, that is, one copy in one chromosome and three copies in the other chromosome; however, a more severe phenotype has been reported in other X-linked gain-of-dose associated with triplications or even quintuplication [16].

No cases with duplication or triplication of the whole functional copy of *PCDH19* gene and DEE9 phenotype have been reported to date. In fact, it is important to note that Patient 2, with a similar duplication of the *PCDH19* gene and neighboring genes in hemizygous state, showed a very different phenotype, with a growth deficit, developmental delay, and ASD but not epilepsy. In the absence of cellular mosaicism, because of being a male, the DEE9 phenotype is not expected. The overlapping region between the duplication of Patient 2 and the triplication of Patient 1 contains the following genes: *PCDH11X*, *NAP1L3*, *FAM133A*, *BRDTP1*, *DIAPH2*, *RPA4*, *PCDH19*, *TNMD*, *TSPAN6*, *SRPX2*, *SYTL4*, *CSTF2*, *NOX1*, *XKRX*, *ARL13A*, *TRMT2B*, *TMEM35A*, and *CENPI*. Among these, only *PCDH19* [1,2,3,4] and *SRPX2* [17] have been demonstrated to cause neurodevelopmental disorder and/or seizures, while a missense variant in *CSTF2* gene has been proposed to cause mild speech delay and learning difficulties in affected males [18]. However, none of these genes has been proven to be pathogenic by duplication or triplication. In fact, as far as we know, no report of another male patient bearing the *PCDH19* duplication has been published. However, the clinical features of patient 2 are variably present in male patients carrying smaller partially overlapping duplications in Xq21.31–q21.32, which does not include the *PCDH19* gene [14,15]. The common region only includes the genes *PCDH11X*, *NAP1L3*, and *FAM133A*, none of which has been associated with a clinical phenotype so far, although *PCDH11X* is thought to play an important role in the development of intellectual disability given its high expression in the nervous system and its involvement in intercellular communication, synaptic plasticity, and verbal ability [19]

Interestingly, it has been recently shown that cortical interneuron migration is affected by altering the PCDH19 dosage by means of overexpression in brain slices and medial ganglionic eminence (MGE) explants. In addition, subtle defects when PCDH19 expression was reduced in MGE explants suggest that the dosage of PCDH19 is important for proper interneuron migration. These findings support that cortical interneuron migration is dependent on a balanced PCDH19 dosage [20]. Similarly, the presumed gain of function reported for some missense variants induces a preferential adhesion and impairment of neuronal development, which advocates for a pathogenic condition of heterotypic overexpressed PCDH19 [9].

A further reinforcement of the pathogenicity of a heterozygous gain of dosage of *PCDH19* comes from the fact that this mechanism has already been described in craniofrontonasal syndrome (CFNS). This is the other X-linked disorder affecting exclusively heterozygous carrier females or males with mosaic pathogenic variants caused by loss-of-function variants in the *EFNB1* gene (OMIM *300035). This gene codifies ephrinB1, part of the Eph/ephrin system, which is a versatile regulator of embryonic morphogenesis by providing positional cues required for the normal morphogenesis of skeletal elements [21]. The pathogenicity of the mosaic expression of this X-linked gene was proven in a study performed with mosaic KO/+ mice, where evidence for reduced the cell mixing between the mosaic areas of the mesenchyme was found [21]. Later, a duplication was proved to be a pathogenic mechanism observed in humans with transcriptional studies as well as in a mouse model [22]. Therefore, it is highly conceivable that a similar mechanism occurs in the *PCDH19* gene, given the clear parallelism between this gene and *EFBN1*, since they both are X-linked genes that cause disease in cases of mosaicism due to a cellular interference mechanism. Furthermore, we consider that the dosage ratio in our patient 1 was three to one, instead of the 2:1 ratio of the usual duplications.

## 5. Conclusions

In conclusion, given the finding of unbalanced triplication in *PCH19*, whose pathogenic mechanism is cellular interference, in a female affected by epileptic encephalopathy compatible with DEE9, we propose that the heterozygous copy-number gain of this gene may cause DEE9, a hypothesis that needs to be further supported by additional evidence.

## Data Availability

The original contributions presented in the study are included in the article; further inquiries can be directed to the corresponding author.
